# Breakthrough of solid tumor treatment: CAR-NK immunotherapy

**DOI:** 10.1038/s41420-024-01815-9

**Published:** 2024-01-20

**Authors:** Wenkang Wang, Yang Liu, Zhen He, Lifeng Li, Senbo Liu, Mingqiang Jiang, Bing Zhao, Meng Deng, Wendong Wang, Xuefang Mi, Zhenqiang Sun, Xin Ge

**Affiliations:** 1 Department of Breast Surgery, The First Affiliated Hospital of Zhengzhou University, Zhengzhou, China; 2 Department of Radiotherapy, Affiliated Cancer Hospital of Zhengzhou University, Henan Cancer Hospital, Zhengzhou, China; 3 Department of Internal Medicine, Affiliated Cancer Hospital of Zhengzhou University, Henan Cancer Hospital, Zhengzhou, China; 4 Biotherapy Center, The First Affiliated Hospital of Zhengzhou University, Zhengzhou, China; 5 Department of Colorectal Surgery, The First Affiliated Hospital of Zhengzhou University, Zhengzhou, China

**Keywords:** Cancer, Tumour immunology

## Abstract

As the latest and most anticipated method of tumor immunotherapy, CAR-NK therapy has received increasing attention in recent years, and its safety and high efficiency have irreplaceable advantages over CAR-T. Current research focuses on the application of CAR-NK in hematological tumors, while there are fewer studies on solid tumor. This article reviews the process of constructing CAR-NK, the effects of hypoxia and metabolic factors, NK cell surface receptors, cytokines, and exosomes on the efficacy of CAR-NK in solid tumor, and the role of CAR-NK in various solid tumor. The mechanism of action and the research status of the potential of CAR-NK in the treatment of solid tumor in clinical practice, and put forward the advantages, limitations and future problems of CAR-NK in the treatment of solid tumor.

## Facts

### What was known before

- This article provides a detailed introduction to the factors that currently constrain the efficacy of CAR-NK in solid tumors, providing reference for the development and design of CAR-NK in the future.
- At present, most of the experiments are about the effect of CAR-NK on hematological tumors. This article focuses on the latest feasibility of CAR-NK therapy in different solid tumors. Provide theoretical basis for expanding the applicability of CAR-NK therapy.
- The author collected ongoing clinical trials of CAR-NK applied to solid tumors, providing ideas for subsequent clinical trial initiators and avoiding duplicate work. At the same time, the latest developments in these experiments can also be monitored.

## Open questions

Due to the recent proposal of CAR-NK therapy, there are currently very few CAR end designs available for reference, especially for solid tumors. It is unclear whether there is a universal design that is effective for all solid tumors or whether there is a specific optimal solution design for each solid tumor. Moreover, the current experimental sample size is generally too small and lacks integration. We need a large number of basic and clinical trials to verify and compare the effects of various CARs. Further validation is needed to determine whether CAR-NK can be widely applied in the treatment of solid tumors in clinical practice in the future.

## Introduction

Under normal circumstances, the immune system can identify and eliminate tumor cells within the Tumor microenvironment (TME). However, to survive and grow, tumor cells employ diverse strategies to suppress the immune system, enabling their survival during different stages of the anti-tumor immune response. This phenomenon, where tumor cells exhibit the described characteristics, is termed ‘immune escape’ [1]. Tumor immunotherapy is a treatment method to control and eradicate tumors by combating immune escape and reinstating the body’s normal anti-tumor immune response. Chimeric antigen receptors (CARs) are fusion proteins, and the CAR structure of CAR-NK cells typically comprises three components: the extracellular antigen-binding region (usually scFv), the spacer and the transmembrane domain, and the intracellular activation domain. Natural Killer (NK) cells, as unique innate immune cells, display rapid and potent cytotoxicity for cancer immunotherapy and pathogen clearance without prior sensitization or antigen recognition [2]. CAR-NK cells are engineered to express CAR through genetic modification, connecting antibodies (or receptors) recognizing surface antigens of target cells (e.g. virus infected cells and cancer cells) with Signaling molecule required to activate immune cells. This modification can counteract inhibitory receptors, thereby enhancing NK cells’ specific killing effect on target cells [3].

For patients with solid tumor who are clinically advanced or extensively metastasized with poor responses to surgical and conventional treatments, CAR-NK cells undoubtedly offer hope. Maximizing the role of CAR-NK in solid tumor is a current challenge in the field of oncology. Successful CAR-NK therapy in solid tumor requires to addressing various difficulties, including designing the optimal CAR structure and genetically modifying the intrinsic inhibitory and activating pathways of NK cells [4]. Current research indicates that the suppressive TME (nutritional deprivation and immunosuppressive state) poses a significant obstacle to the effective application of CAR-NK in solid tumors. However, the functional state and damage mechanism of NK cells within the TME remain unknown [5]. The TME can induce severe dysfunction in cytotoxic immune cells, and the extent of impaired cytotoxicity in NK cells within the TME of cancer patients is closely linked to the prognosis of various cancers. Despite this, the underlying mechanisms of NK cell dysfunction in the TME are not yet fully understood. This article addresses the challenges posed by the TME to the application of CAR-NK in solid tumors. It delves into the construction process of CAR-NK, exploring the impacts of hypoxia, metabolic factors, NK cell surface receptors, cytokines, and exosomes on the efficacy of CAR-NK in solid tumors. The mechanisms governing the functioning of CAR-NK in various solid tumors and the current research status of CAR-NK’s clinical potential for treating solid tumors are comprehensively reviewed.

## Tumor immunotherapy

Nonspecific immunity comprises mucosal epithelial cells, phagocytes, and natural killer cells, demonstrating characteristics such as a wide range of action, rapid response, relative stability, and heritability. An effective immune response aims to eliminate malignant cells or inhibit their growth [6]. Despite the innate effectiveness of nonspecific immunity, cancer cells can elude immune surveillance by impeding antigen presentation, employing negative regulation, and recruiting immunosuppressive cell populations [7, 8]. This leads to the impairment of immune cell effector functions and the failure of anti-tumor immune responses. In response to this challenge, immunotherapy emerged, with the principle of enhancing or improving the anti-cancer abilities of immune cells to prevent immune escape by cancer cells. Currently, immunotherapy falls into five categories: targeted antibody [9], adoptive cell therapy [10], oncolytic virus [11], cancer vaccine [12] and immunomodulator [13]. Tumor immunotherapy offers irreplaceable advantages: Firstly, it activates the body’s immune system, restoring immune function and persistently eliminating tumor cells; Secondly, it reconstructs and enhances overall immune function, effectively preventing tumor recurrence and metastasis with systemic effects.; Thirdly, it improves the body’s immune ability, thoroughly eliminating residual tumor cells and micro-metastasis lesions; Fourthly, Immunotherapy has a broad range of applications, suitable for solid malignant tumors, hematological malignancies, etc., especially beneficial for malignancies with multiple lesions or extensive metastasis. For patients with advanced cancer who have lost the opportunity for surgery, are in poor physical condition, or cannot tolerate high-dose radiotherapy or chemotherapy or are insensitive and resistant to chemotherapy, biological therapy alone emerges as a significant option. This approach not only significantly improves symptoms and enhances the quality of life but also prolongs survival time [14, 15]; Importantly, the side effects are relatively mild, attributed to the use of molecular targeting technology, which selectively impacts tumor cells without affecting normal cells and ensures higher safety [16, 17]. Among the various biological therapy modalities, adoptive Cell therapy (ACT) has garnered increasing attention. ACT involves the collection of human autoimmune cells, which are cultured in vitro to expand their numbers or enhance their targeted killing function. Subsequently, these modified cells are infused back into the patient’s body to combat pathogens, cancer cells, or mutated cells in the blood and tissues. ACT encompasses various approaches, including Tumor Infiltrating Lymphocyte (TIL) therapy, Engineered T Cell Receptor (TCR) therapy, CAR-T therapy, and CAR-NK therapy.”

## CAR-NK therapy

The most promising application in tumor immunotherapy lies in CAR-engineered immune cells within adoptive cell therapy. CAR, an artificially modified fusion protein, possesses an extracellular antigen recognition domain and several genetically modified intracellular signaling domains. The initial attempt at this technology, the first-generation CARs, had a basic structure comprising an extracellular single-stranded variant (scFv) responsible for identifying cancer cells and an intracellular signal transduction domain (CD3 ζ Chain) crucial for cell activation.The intracellular domains of the second and third generation CARs typically include one or two co-stimulatory signaling molecules, such as 4-1BB (CD137), CD28, CD27, OX40 (CD134), inducible T cell co-stimulatory factor (ICOS), or regulatory subunit I anchoring disruptor (RIAD). These enhance cell activation, proliferation, and survival time. The fourth generation CARs, also known as precision CARs or armored CARs, release immune modulators upon reaching the tumor microenvironment. They target the tumor microenvironment, release immune regulatory factors, and attract/activate more immune cells to attack tumor cells [18]. CAR-NK achieves gene transduction through various methods, including retroviruses, lentiviruses, electroporation, liposomes, and DNA transposons [19]. Retroviral vector transduction has two disadvantages: one is that the virus can be inserted into the genome, which may lead to cellular cancer; the other is that retroviral transduction can inhibit the viability of primary NK cells. Lentivirus has low genotoxicity and insertional mutagenesis. Nonetheless, the transduction efficiency of lentivirus in primary NK cells is relatively low, and multiple rounds of transduction are usually required. Electroporation and liposome transfection can also effectively introduce exogenous genes into NK cells, and the transferred genes express quickly, the level of apoptosis is low, and the inter-individual variability is small. However, the exogenous DNA transduced by electroporation and liposomes will not integrate into the genome of the target cells, so the expression of the transgene is relatively short-lived. DNA transposons are mobile DNA elements that can be efficiently transposed between vectors and chromosomes through a “cut and paste” mechanism, resulting in CAR-iPSC-NK cells stably expressing CAR molecules. Compared with viral vectors, these transposon systems have several advantages, such as low immunogenicity, high biosafety, low production cost, and the ability to transduce large gene fragments longer than 100 kb. However, the transposon system still needs to overcome the disadvantages of low transduction efficiency and NK cell death caused by electroporation of plasmid DNA. The immunosuppressive effect of the microenvironment is the biggest obstacle to the application of CAR-NK in solid tumor. In view of this, the design of CAR-NK for the treatment of solid tumor should choose a scheme that can bypass or improve the TME to maximize the efficacy [20, 21].

Compared with CAR-T therapy, CAR-NK therapy has its unique advantages and characteristics due to the replacement of carrier cells from T to NK cells. NK cells have cytotoxic activity and function most similarly to CD8 + T cells. The low risk of rejection of NK cells allows NK cells in CAR-NK to be generated from a variety of sources. NK cells originate from the spleen, liver, secondary lymphoid organs, thymus, intestine, tonsil, and uterus. Unlike B cells and T cells, NK cells are a type of lymphocytes that can non-specifically kill tumor cells and virus-infected cells without prior sensitization. NK cells mainly kill target cells through three mechanisms: Firstly, direct killing of target cells by releasing cytoplasmic granules containing perforin and granzymes. Secondly, The release of cytokines, such as IFN-γ, TNF-α, etc., induces tumor cell apoptosis through the interaction with the corresponding receptors on the surface of tumor cells. Thirdly, The Fc receptor CD16 binds to the Fc region of the antibody, which can trigger antibody-dependent cell-mediated cytotoxicity (ADCC) to kill cells. These diverse killing mechanisms are the basis for the dual anti-tumor activity of CAR-NK cells [21]. When designing CAR-NK CARs, the following aspects can be considered: 1.Autocrine cytokines such as IL-2 and IL-15 can enhance NK cell toxicity and promote NK cell proliferation [22]; 2.Change the metabolic composition of tumors or modify gene expression programs in immune cells to protect them from the invasion of inhibitory metabolites in TME [23]; 3.Tumors can evade immune surveillance and improve metabolic adaptability and anti-tumor activity by blocking inhibitory immune checkpoint proteins such as PD-L1, LAG-3, and TIGIT [24**–**26]; 4.Equip NK cells with stable ectopic chemokine receptors to enhance their ability to enter and penetrate tumors [27]. CAR-NK therapy is receiving increasing attention due to its safety and efficacy.

## Influence of hypoxia and metabolic factors on the efficacy of CAR-NK in solid tumor

**Fig. 1:**
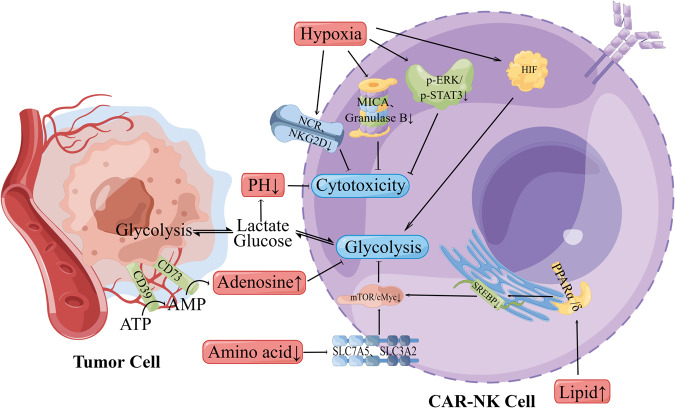
Mechanisms of hypoxia and metabolic factors affecting the effect of CAR-NK. Under hypoxic conditions, the phosphorylation levels of ERK and STAT3 were reduced, the activating receptors of CAR-NK cells were inhibited, and MICA and granzyme B were reduced. Although the glycolysis of CAR-NK cells could be activated through HIF, overall Caused a decrease in the cytotoxicity of CAR-NK cells. After glycolysis became the main metabolic mode, lactic acid increased significantly, resulting in a decrease in the pH of TME and a decrease in the cytotoxicity of CAR-NK cells. Tumor cells consume amino acids and produce lipids, which can inhibit the glycolysis of CAR-NK cells and affect the energy metabolism of CAR-NK cells. By Figdraw.

Hypoxia refers to the pathological process involving abnormal changes in the metabolism, function and morphological structure of tissues due to insufficient oxygen supply or disorders in oxygen utilization within tissues. The hypoxic TME can induce aberrant angiogenesis, reprogram energy metabolism, facilitate immune evasion, activate invasion and metastasis, induce pro-tumor inflammation, maintain proliferative signals, and cause genomic instability [28]. Therefore, adequate attention should be given to the impact of the hypoxic TME on the efficacy of CAR-NK. This section elucidates the mechanisms and corresponding strategies regarding the effects of hypoxia and metabolic factors on the efficacy of CAR-NK (Fig. 1). Throughout tumor development and progression, cancer cells and stromal cells often face challenges in obtaining nutrients and oxygen due to poorly developed, disorderly distributed, and easily leaky tumor blood vessels. Most solid tumors exhibit areas of permanent or temporary hypoxia [29]. Hypoxia leads to the upregulation of hypoxia-inducible factors (HIFs) composed of oxygen-sensitive alpha subunits (HIF-1α, HIF-2α, and HIF-3α) and constitutively expressed beta subunits (HIF-1β). Studies indicate that hypoxia can reduce the phosphorylation levels of ERK and STAT3 in a protein tyrosine phosphatase 1 (SHP-1)-dependent manner, impairing the cytotoxicity of NK cells [30]. Low O2 concentration hinder the expression and function of activated receptors NKp44, NKp46, NKp30, and NKG2D in NK cells [31], while reducing MICA (Major histocompatibility complex class Related chain A) [32]. Furthermore, hypoxia increases the autophagy level of tumor cells, leading to enhanced degradation of granzyme B [33]. Despite promoting NK cell glycolysis, hypoxia ultimately results in decreased NK cell toxicity.

The hypoxic microenvironment of tumor cells limits cellular sources, resulting in a substantial accumulation of extracellular adenosine triphosphate (ATP). Tumor cell membrane nucleases CD39 and CD73 act on ATP, converting it into adenosine (ADO). ADO, in turn, inhibits NK cell maturation through A2AR, compromising the anti-tumor immune function of NK cells [34, 35]. Simultaneously, ADO affects vascular endothelial cells and stromal cells, promoting the formation of TME neovascularization and creating favorable conditions for tumor metastasis [36]. Studies have shown that CD73 expression is upregulated during the growth of breast cancer and sarcoma [37]. In hypoxic conditions, glycolysis in the TME generates significant amounts of lactic acid. Lactate inhibits the upregulation of nuclear factor of activated T cells (NFAT), which in turn reduces NK cell IFN-γ production. Lactic acid (low pH environment) also increases the number of myeloid-derived suppressor cells (MDSC) and induces apoptosis [38, 39]. Furthermore, Brand et al. [40] also showed that lactate uptake by mouse NK cells leads to intracellular acidification and impaired energy metabolism, severely inhibiting their antitumor activity. NK cell metabolism heavily relies on the mammalian target of rapamycin complex 1 (mTORC1) [41], a nutrient/metabolic sensor activated by amino acids, coordinating protein synthesis. Inhibition of mTORC1 leads to decreased NK cell activity [42]. Phosphorylation of MTOR is associated with NK cell maturation in bone marrow and spleen [43]. Amino acids, especially glutamine, entering cells through transporter SLC7A5/SLC3A2, and direct regulation of c-Myc, are crucial pathways in NK cell metabolism [44]. c-Myc is involved in regulating the expression of glucose transporters and glycolytic enzymes. Additionally, sterol regulatory element-binding protein (SREBP), a transcription factor, regulates glycolysis and oxidative phosphorylation in NK cells. Tumor cells triggering PPARα/δ signaling by increasing lipid metabolism and fatty acid exposure inhibit SREBP, diminishing cytokine production and NK cell cytotoxic activity [45].

Currently, addressing hypoxia involves controlling HIF levels. Bortezomib, the first discovered proteasome inhibitor, can inhibit HIF-1 transcriptional activity [46]. The mTOR signaling inhibitor temsirolimus has demonstrated the ability to block HIF-1α translation [47]. Normalizing tumor vasculature can enhance the response to hypoxia, as hypoxic tumors overexpress pro-angiogenic proteins like VEGF, resulting in abnormal vascular networks that promote immune escape and reduce immunotherapy efficacy. Administering anti-angiogenic drugs targeting VEGF or its receptors can normalize blood vessels. However, monotherapy with anti-angiogenic drugs may exacerbate tumor hypoxia, leading to treatment resistance and worsened patient outcome [48]. Combining immunotherapy with vascular normalization appears promising for improving patient outcomes. Studies by Michelet’s group suggest that targeting tumor therapy with rapamycin, inhibiting mTORC1 and glycolysis, may effectively limit tumor growth by restraining Warburg’s glycolytic metabolism in tumor cells [49].

## Effect of NK cell surface receptors on the efficacy of CAR-NK in solid tumor

### NKG2D/NKG2DLs

NKG2D, encoded by the Klrk1 gene, serves as an activating receptor on the surface of NK cells. In humans, NKG2D forms a complex with the adapter protein DAP10, transmitting downstream signals through the charge in its transmembrane domain. DAP10, also known as KAP10, is a small transmembrane adapter protein with a YINM sequence in its cytoplasmic tail [50]. It binds the p85 subunit of PI3K and Grb2, closely associated with Vav1. NKG2D activates NK cells and delivers co-stimulatory signals to CD8 + T cells [51]. The molecular structure of NKG2D allows it to bind many structurally distinct MHC I-like ligands (NKG2DLs) [52]. The expression of NKG2DLs is upregulated during malignant transformation, oxidative stress, and viral infection. MICA/B, mainly MICA, MICB, and ULBP1-6 in humans [53], is found in gastrointestinal epithelial tumors, as well as lung, breast, kidney, ovary, and prostate tumors [54]. MICA/B contains α1, α2 and α3 domains, which are encoded by genes in MHC with up to 80% homology. It is commonly found in gastrointestinal epithelial tumors [55], and can also be found in lung, breast, kidney, ovary, and prostate tumor [56]. ULBP1-6 contain α1 and α2 domains and can be transmembrane-linked (ULBP4 and 5), glycosylphosphatidylinositol (GPI)-linked (ULBP1-3, 6) or both (ULBP2 and 5) [51]. Studies have demonstrated that the activation signal mediated by NKG2D binding to ligand can bypass the inhibitory receptor-induced signal to activate NK cells [57]. NKG2D is a multifunctional receptor that can directly bind to a variety of ligand molecule families expressed on the surface of target cells without antigen presentation, thereby activating or co-stimulating immune effectors [58]. This activation is followed by the release of cytolytic proteins like perforin and granzymes, which mediate killing in tumor cells. The NKG2D-mediated immune response plays a crucial role in tumor surveillance, and the NKG2D pathway can regulate tumor initiation and progression, which is essential for ensuring the efficacy of CAR-NK in solid tumors [53].

The expression of NKG2D in human NK cells can be upregulated by IL-15, IL-10, IL-12, TNF-α and IFN-α [59]. The downregulation of NKG2D may be due to the production of soluble NKG2DLs by tumor cells. Many results suggest that high concentrations of soluble NKG2DLs may inhibit tumor immunity and NK cell activity through downregulation of NKG2D expression or proteolytic shedding of MICA/B. The upregulation of soluble NKG2DLs expression is associated with breast cancer Lymph node metastasis is associated with poor prognosis in solid tumors such as melanoma, neuroblastoma, prostate cancer, and kidney cancer [56, 60–63]. In addition to soluble NKG2DLs, cytokines in the TME are also involved in NKG2D-mediated tumor escape mechanisms. The pro-inflammatory cytokines IFN-γ and TGF-β downregulate the expression of MICA and ULBP and inhibit NKG2D-mediated NK cell activation [64**–**66].

It should be noted that recent studies have found that NKG2D can enhance NK cell-mediated ADCC in a synergistic manner, which can increase the anti-tumor activity of CAR-NK and serve as a complement to the way CAR kills solid tumor [67]. Guo et al. [68] designed chimeric PD1-NKG2D receptors containing the NKG2D hinge region and 4-1BB co-stimulatory domain, showed stable surface expression, and mediated the in vitro cytotoxicity of NK92 cells against various tumor cells. Professor Xiao [69] constructed CAR-NK by fusing the extracellular domain of NK cell receptor NKG2D with DAP12, having a significant effect on mice with solid tumor. Three patients with metastatic colorectal cancer were subsequently treated with local infusions of CAR-NK cells. Reduced ascitic fluid production and a significant reduction in the number of tumor cells in ascites samples were observed in the first two patients treated with low-dose CAR-NK cell intraperitoneal infusions. Rapid tumor regression in the hepatic region was observed by Doppler ultrasound imaging and complete metabolic response in the treated liver lesions was confirmed by positron emission tomography (PET)-computed tomography (CT) scans.

### Killer cell immunoglobulin-like receptor (KIR)

KIR is an Ig-like receptor molecule that can bind to certain HLA-I class molecules, serving as a key regulator of NK cell function. It is categorized into KIR2D (two domains) and KIR3D (three domains) based on the number of extracellular Ig-like domains. In KIR2D/3D, the cytoplasmic region containing the ITIM motif has a longer amino acid sequence, known as KLR2DL/3DL, acting as an NK cell inhibitory receptor. The other part of the cytoplasmic region containing the ITAM motif has a shorter amino acid sequence, known as KIR2DS/3DS. The transmembrane region contains a positively charged lysine, forming an NK cell killing activated receptor when combined with negatively charged aspartic acid in the transmembrane region and the DAP-12 homodimer containing ITAM in the cytoplasmic region [70]. NK cells undergo trogocytosis, a biological process involving the gnawing off of a part of the cell membrane and its surface molecules from antigen-presenting cells through the immune synapse. This process allows NK cells to transfer cell membrane surface substances from target cells to effector cells. CAR activation in NK cells facilitates the transfer of CAR cognate antigens from tumors to NK cells. However, this can lead to a decrease in tumor antigen density, weakening CAR-NK cell binding to targets. Additionally, it may induce self-recognition, sustained CAR-mediated binding, self-killing, and low reactivity in NK cells expressing granulocyte antigens (NKTROG+) [71, 72]. This phenomenon can be counteracted by a dual-CAR system that combines an activating CAR directed against a cognate tumor antigen and an NK self-recognition inhibitory CAR that delivers a “don’t kill me” signal to NK cells upon contact with TROG+. This system prevents granulocyte antigen-mediated cannibalism while preserving activating CAR signaling against tumor antigens and leads to enhanced CAR-NK cell activity. Li [73] reported aCAR-mediated exocytosis, which contributed to the reduction of target antigen density, as well as NK cell cannibalism and hyporesponsiveness. They used an antigen-specific inhibitory KIR-based receptor (iCAR) that successfully inhibited aCAR-mediated TROG antigen-induced NK cell cannibalism, preventing aCAR NK cells from producing TROG antigen-induced immunomodulatory consequences while retaining the ability to express the same antigen key effector functions of tumor cells. This dynamically modulated AI-CAR signaling may find useful applications to improve the in vivo persistence and therapeutic efficacy of a range of adoptive NK cell therapies.

### DNAX accessory molecule 1(DNAM-1)

Human DNAM-1 (CD226) is a type I transmembrane glycoprotein, approximately 65 kilodaltons (kDa) in size. It consists of an 18 amino acid (aa) leader sequence, a 230 amino acid extracellular domain with two Ig-like C2 group domains, a 28 amino acid transmembrane domain, and a 60 amino acid cytoplasmic domain containing two residues (Tyr322 and Ser329) [74]. DNAM-1 serves as an activating receptor that triggers NK cell-mediated cytotoxicity upon interaction with the ligands CD155 and CD112 [75]. Notably, the PVR/CD155 and Nectin-2/CD112 ligands of DNAM-1 are mainly expressed on solid tumor cells, especially those of epithelial and neuronal orig [76, 77], and in normal tissue cells very little expression. Focaccetti’s team [78] found that FL-DNAM-1-CD3z engineered NK cells combined with immunomodulatory drugs such as Nutlin3a could represent a new immunotherapeutic approach for the treatment of p53 dysfunctional neuroblastoma. Given that DNAM-1 ligands are expressed in many solid tumor [79], this treatment approach proves effective not only for neuroblastoma but also for colorectal cancer, breast cancer, ovarian cancer, lung cancer, pancreatic cancer, and other solid tumors exhibiting p53 dysfunction [80].

### Natural cytotoxicity receptor (NCR)

**Fig. 2:**
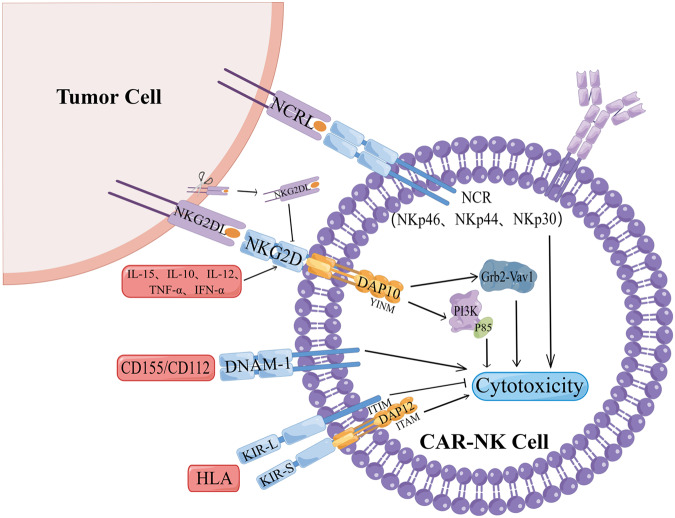
The mechanism of NK cell surface receptors affecting the effect of CAR-NK. After the NKG2D receptor on the surface of CAR-NK cells binds to NKG2DL, it can form a complex with DAP10 to activate the P85 subunit of Grb2-Vav1 and PI3K, thereby enhancing the cytotoxicity of CAR-NK cells. IL-15, IL-10, IL-12, TNF-α, and IFN-α in the TME activate NKG2D receptors, whereas soluble NKG2DL (sNKG2DL) inhibits NKG2D receptors. KIR receptors can be divided into activating KIR S (ITAM) and KIR L (ITIM) according to the different motifs contained in the amino acid sequence of the cytoplasmic region. Combination of DNAM-1 with ligands CD155 and CD112 can enhance CAR-NK cytotoxicity. NCR is a group of CAR-NK cell surface activating receptors, including NKp46, NKp44 and NKp30. By Figdraw.

NCR is a group of surface activated receptors on NK cells, including NKp46 (NCR1, CD335), NKp44 (NCR2, CD336), and NKp30 (NCR3, CD337). All three are members of the immunoglobulin superfamily (IgSF), but have no homology with each other [81] and typically play a killing role when KIRs lose their ability to recognize themselves. NKp46 and NKp30 are expressed on the surface of all NK cells. The extracellular region of NKp46 contains two Ig-like domains, and the extracellular region of NKp30 has only one V-type domain. The cytoplasmic regions of NKp46 and NKp30 are relatively short, and the transmembrane regions both contain positively charged arginine. When they interact with the transmembrane region containing negatively charged Aspartic acid, the cytoplasmic region containing ITAM motif CD3 ζζ When homologous dimers are not covalently bound, they are able to transduce activation signals. In contrast, NKp44 is exclusively expressed on the surface of activated NK cells, serving as a specific marker. Its extracellular region features a single V-type domain. Unlike NKp46 and NKp30, the cytoplasmic region of NKp44 lacks the ITAM motif. However, its transmembrane region contains positively charged lysine, allowing non-covalent binding with negatively charged aspartic acid in the transmembrane region and the DAP12 homodimer containing the ITAM motif in the cytoplasmic region, thereby transmitting activation signals. Studies have shown that NKp30 downregulation is associated with neuroblastoma metastasis and chemotherapy resistance [82]. At present, there is a lack of data on the application of NCR in CAR-NK targeting solid tumor, which requires further verification (Fig. 2).

## Effect of cytokines (CK) on the efficacy of CAR-NK in solid tumor

### TGF-β

Transforming growth factor-β (transforming growth factor-β, TGF-β) belongs to a group of newly discovered TGF-β superfamily that regulates cell growth and differentiation. In addition to TGF-β, this family also includes activins, inhibitors, Mullerian inhibitor substances (MIS) and bone morphogenetic proteins (BMPs) [83]. TGF-β It is mainly produced by tumor cells, regulatory T cells (Tregs) and myelogenous Sexual inhibition cells (MDSCs) in TME. It is closely related to the poor prognosis of lung cancer, pancreatic cancer, gastric cancer, colorectal cancer, breast cancer and liver cancer, and almost all the immune system cells express TGF- β Receptors (TGF-βR) [84**–**89]. TGF-β is secreted as an inactive dimer that requires processing by a different mechanism for activation. Active TGF-β binds to a tetrameric receptor consisting of two TGF-βRI chains and two TGF-βRII chains. Upon binding to TGF-β, type II receptors phosphorylate type I receptors, which then propagate the signal through phosphorylation of the transcription factor Smad2/3 [90]. This complex then moves to the nucleus, binds to Smad4 and other cofactors and mediates a decrease in NK cytotoxicity.

TGF-β inhibits NK cell activation and function by downregulating NK cell-activating receptors like NKG2D, DNAM1, and NKp30, as well as metabolic pathways such as mTOR/c-Myc. It directly impairs NK cell function by downregulating the activation of NK receptors, particularly NKG2D and NKp30, or inhibiting the mTOR pathway, aiding tumor cell immune evasion [91]. TGF-β production increases during tumor growth and malignant progression, and selectively suppresses the expression of MICA, ULBP2, and ULBP4, while MICB, ULBP1, and ULBP3 are unaffected [65]. Highly enriched in the solid tumor microenvironment, TGF-β has a significant immunosuppressive effect. It enhances the expression of CXCR3 and CXCR4 while inhibiting the expression of CX3CR1 in human NK cells, hindering NK cell egress from the bone marrow and inhibiting NK cell maturation [92]. Cells express a variety of antioxidant proteins, such as superoxide dismutase (SOD) and catalase, which can convert reactive oxygen species (ROS) into water and oxygen. ROS can be produced under steady-state conditions and participate in various biological processes such as redox signaling pathways and apoptosis. Excessive ROS can damage various cellular components such as DNA and protein of NK cells. Excess ROS may also lead to abnormal NK cell signaling through oxidative modification of redox-sensitive signaling proteins such as MAPK, HIF, or NFkB. Michaeloudes et al. [93] discovered that TGF-β can also inhibit NK cell activity by stimulating ROS generation. The present study showed that co-transduction of NK cells with B7H3 CAR and transforming growth factor-β dominant negative receptor (DNR) preserved cytolytic function in the presence of exogenous transforming growth factor-β. This novel DNR and CAR co-expression strategy may be a promising approach to treat refractory CNS tumors like GBM [94].

### Interleukin (IL)

Interleukins are pivotal mediators of inflammation, governing various aspects of NK cell biology. NK cells express cytokine receptors early in their development [95] and require signaling through a common gamma (γc) chain for development, homeostasis and functionalization. The γc chain, a 40 kDa type I transmembrane glycoprotein, is the signaling subunit of interleukins. Both IL-2 and IL-15 can signal through a complex consisting of γc and IL-2Rβ chains [96], activating STAT1 and STAT5 through JAK-1 and JAK-3, respectively [97]. Activated NK cells express IL-2Rα (CD25), which significantly enhances their affinity to IL-2, promoting NK cell proliferation and the production of lytic molecules such as perforin and granzyme B [98]. Trans-presentation of IL-15 from IL-15Rα to IL-15Rβ/IL-2Rγ complexes on NK cells initiates cell proliferation and transcriptional reprogramming. The IL-12 heterodimeric cytokine family includes IL-12, IL-23, IL-27 and IL-35. IL-12, composed of p40 and p35 α and β subunits, binds to the IL-12 receptor (IL-12R) complex IL-12Rβ1/IL-12Rβ2, and IL-12R signaling is regulated by tyrosine kinase-2/JAK- 2 transmits and activates the transcriptional regulator STAT4 [99]. Li [100] and Silvestre [101] demonstrated that the fourth- generation CD19-targeted CAR (CAR.19) co-expressing IL-15 or IL-15/IL-15Rα significantly enhanced NK-92 cell proliferation, proinflammatory cytokine secretion, and cytotoxic activity against cancer cell lines in vitro and in a xenograft mouse model.

**Fig. 3:**
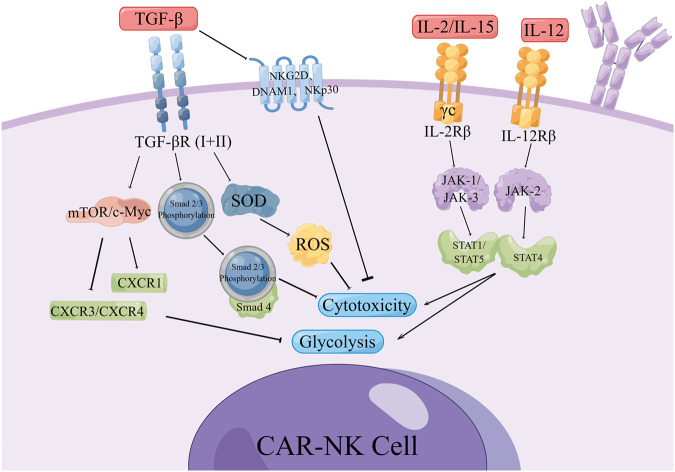
Mechanisms of cytokines affecting the effect of CAR-NK. TGF-β in the TME binds to TGF-βR on CAR-NK cells to phosphorylate the transcription factor Smad2/3 complex, which then moves to the nucleus, binds to Smad4 and other cofactors, and mediates NK Decreased cytotoxicity; it can also reduce CAR-NK glycolysis and affect energy metabolism by down-regulating mTOR/c-Myc. Soluble TGF-β can also directly inhibit the activating receptors on the surface of CAR-NK such as NKG2D, DNAM1 and NKp30, thereby reducing the cytotoxicity of CAR-NK. Both IL-2 and IL-15 can signal through a complex composed of γc and IL-2Rβ chains, and IL-12 can bind to IL-12R to enhance glycolysis and cytotoxicity of CAR-NK cells. By Figdraw.

Current data support the positive role of interleukins in CAR-NK cell therapy for solid tumors. Pérez-Martínez [102] found that allogeneic IL-15-stimulated CAR-NK cells may be feasible and safe in children with refractory solid tumors. Studies have demonstrated that matching CAR-NK cells with tumor-secreted chemokines (such as IL-8 and receptor CXCR1) enhances tumor migration and invasion [103]. NK cells, lacking peroxiredoxin-1 (PRDX1), can benefit from IL-15, which upregulates PRDX1 expression and protects NK cell function. Klopotowska et al. [104] engineered PRDX1-overexpressing PD-L1-CAR NK cells, displaying potent antitumor activity against breast cancer cells under oxidative stress (Fig. 3).

## The effect of exosomes on the efficacy CAR-NK in solid tumor

Exosomes are vesicles (EVs) (30–150 nm) that contain complex RNA and proteins. In 1987, Johnstone et al. [105] discovered vesicles released by reticulocytes during in vitro culturing, coining the term ‘exosomes’. A variety of cells can secrete exosomes under normal and pathological conditions, primarily derived from multivesicular bodies formed by lysosomal particle invagination. These vesicles are released into the extracellular matrix after the fusion of the multivesicle’s outer membrane with the cell membrane. All cultured cell types secrete exosomes, which can eventually enter the circulation, entering blood, saliva, urine, cerebrospinal fluid, and breast milk. Exosomes carry diverse proteins, including membrane transport-related proteins (RAB GTPases, annexins, Flillins, ALIX, and TSG101), tetrapeptides (CD9, CD63, CD81, HSP60, and HSP90), nucleic acids (mRNA, miRNA, lncRNA, circRNA), and lipids (sphingomyelin, phosphatidylserine, phosphatidylinositol, phosphatidic acid, ceramide, and cholesterol) [106]. They mediate cell communication and genetic information transmission, playing a crucial role in disease diagnosis and prognosis.

Tumor-derived exosomes (TDEs) contain diverse molecular components, including lipids, membrane-associated proteins, long non-coding RNAs (lncRNAs), and miRNAs, which are involved in several processes of tumor formation and invasion, including angiogenesis, proliferation, and Growth, metastasis and immune escape. Numerous studies indicate that TDEs hinder anti-tumor immunity by impairing the function of dendritic cells (DC), NK cells, and T cells. Among these, NK cells, considered the first line of defense against malignant cell transformation, can have their function inhibited by tumor cells through various mechanisms, with tumor exosomes playing a key role in NK cell dysfunction. TDEs can be taken up by NK cells through various mechanisms, including plasma membrane fusion, endocytosis, phagocytosis, micropinocytosis, and lipid raft-mediated internalization. They induce downstream signaling through receptor–ligand interactions, downregulating NK cell antitumor activity [107]. Tumor exosomes also interfere with NK cell recruitment, migration, proliferation, survival, cytolytic activity, cytokine production, and receptor expression [108]. There is evidence that exosomes from clear cell renal cell carcinoma evade innate immune surveillance by activating the TGF-β/SMAD pathway, thereby inhibiting natural killer cell function [109]. Zhao [110] demonstrated that exosomes from pancreatic ductal carcinoma could downregulate the expression of NK cell activating receptors, leading to NK cell dysfunction. Interestingly, several studies [111, 112] demonstrated that IL-15 could reverse the suppression of NKG2D expression by tumor exosomes and protect NK cells from the inhibitory effect of exosome-associated TGF-β. At present, it has been confirmed that CAR-T EVs can maintain the activity of CAR-T cells, and has a positive effect on hematological tumors and solid tumor [113]. CAR-NK is expected to have a similar effect, waiting for further research.

## Attempts of CAR-NK in different solid tumor

**Fig. 4:**
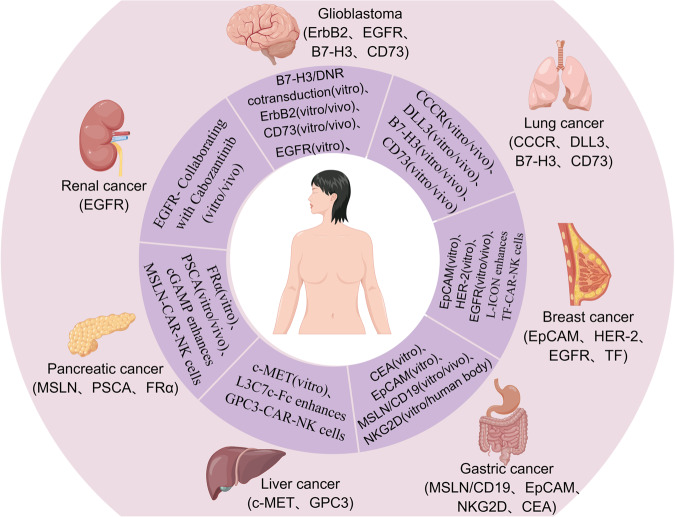
Schematic diagram of CAR-NK exploration experiments in different solid tumors. In brackets are valuable targets that have been discovered so far. And marked the research progress of each solid tumor. By Figdraw.

The exploration of CAR-NK therapy in solid tumors is acknowledged to be in its early stages, with an increasing number of clinical studies being conducted each year. While CAR-NK cells possess unique advantages, they are not without challenges. The hurdles include issues related to cell persistence, overcoming the immunosuppressive microenvironment, and optimizing transduction efficiency, among others. This recognition sets the stage for ongoing research and development to address these challenges and further enhance the effectiveness of CAR-NK therapy in the treatment of solid tumors (Fig. 4).

### Glioblastoma

Recently, gratifying results have been obtained regarding the inhibitory effect of CAR-NK on glioblastoma. The research by Wang et al. [114] indicates that that the development of multifunctional genetically engineered human NK (CD73. mCAR pNK) cells can generate effective anti GBM activity due to the tumor heterogeneity and multiple immunosuppressive characteristics of GBM TME. Targeting autophagy as an immunomodulator promotes the homing of effector CAR-NK cells to GBM tumor sites, while reprogramming the GBM TME to be more sensitive to CAR-based targeting. Zhang [115] demonstrated that growth factor receptor tyrosine kinase ErbB2 (HER-2)-specific NK-92/5.28.z (second-generation CAR) Potent and selective antitumor activity against GBM cells in vitro and in orthotopic GBM xenograft models, as well as cure and induction of endogenous antitumor immunity after NK-92/5.28.z treatment in immunocompetent mice. Murakami [116] designed a novel CAR-NK cell line (CAR-KHYG-1, second-generation CAR) specifically for tumors expressing epidermal growth factor receptor variant III (EGFRvIII). Results showed that EvCAR-KHYG-1 inhibits GBM cell growth through apoptosis in a specific manner expressing EGFRvIII. Chaudhry94 co-transduced CB-derived NK cells with B7-H3 CAR and TGF-β dominant-negative receptor (DNR). This second-generation CAR showed potent cytolytic activity against GBM cells in vitro. Moreover, human leukocyte antigen G (HLA-G), identified as an immune checkpoint protein (ICP), is newly expressed in a majority of tumor cells. JAN [117] found that HLA-G CAR-transduced NK cells exhibited robust cytolytic effects against breast, brain, pancreatic, and ovarian cancer cells in vitro. Furthermore, these cells demonstrated a significant reduction in xenograft tumor growth in an orthotopic mouse model, leading to prolonged median survival. This approach holds promise for future applications in CAR-NK therapy for a range of solid tumors.

### Lung cancer

Lu [118] developed a chimeric co-stimulatory transition receptor (CCCR) consisting of PD1 extracellular domain, NKG2D transmembrane and cytoplasmic domain, and NKG2D cytoplasmic domain 4-1BB. This receptor has the capacity to convert negative PD1 signals into activation signals, effectively reversing the immunosuppressive effect of PD1. In a lung cancer xenograft model, CCCR-NK92 cells(third generation CAR) demonstrated significant inhibition of tumor growth. It’s noteworthy that in clinical application. Dr. Zhang et al. [119] reported that this regimen may cause the occurrence of cytokine release syndrome (CRS), and Clinicians should be vigilant regarding this side effect during CAR-NK treatment.

For non-small cell lung cancer, Liu [120] engineered DLL3-specific NK-92 cells and explored their potential in treating SCLC. Co-culture of the DLL3 SCLC cell line with DLL3-CAR NK-92 cells exhibited significant in vitro cytotoxicity and cytokine production. DLL3-CAR NK-92 cells induce tumor regression in an H446-derived lung metastatic tumor model at a favorable safety threshold. Potent antitumor activity of DLL3-CAR NK-92 cells was observed in a subcutaneous tumor model of SCLC. Furthermore, distinct tumor-infiltrating DLL3-CAR NK-92 cells were detected in DLL3 SCLC xenografts. In a study by Yang [121], NK-92MI cells carrying an anti-B7-H3 CAR(second-generation CAR) effectively restricted the growth of transplanted non-small cell lung cancer in mice, significantly prolonging the survival time compared to unmodified NK-92MI cells. The secretion of perforin/granzyme B and the expression of CD107a were notably elevated in anti-B7-H3 CAR-NK-92MI cells. Chambers et al. [122] designed a CAR-NK targeting the CD73 adenosine axis by blocking CD73 enzymatic activity. This approach not only impairs adenosinergic metabolism driven by hypoxic ATP uptake by cancer cells in an NSCLC model but also induces tumor stasis. It promotes NK cell infiltration into CD73 tumors and enhances intratumoral activation.

### Breast cancer

Epithelial cell adhesion molecule (EpCAM), a pan-epithelial differentiation antigen, is expressed across various cancers and possesses endogenous oncogenic potential. Effector cells co-expressing CAR and IL-15(forth-generation CAR) exhibit effectiveness without exogenous cytokines, enabling continued proliferation under EpCAM conditions. These cells demonstrate highly selective killing activity against EpCAM-expressing breast cancer cells. This strategy facilitates rapid isolation and sustained expansion of relocated NK cells, enhancing their potential clinical utility [123]. For HER-2+ breast cancer, data have shown that the expression of HER-2 CAR in NK cells from healthy donors and breast cancer patients can effectively enhance its anti-tumor function against various HER-2-expressing cancer cells, and compared with MHC class I expression is irrelevant. Importantly, HER-2 CAR-NK cells exhibit enhanced cytotoxicity to tumor targets compared to donor-matched HER-2 CAR-T cells. Notably, HER-2 CAR-NK cells maintain high cytotoxic function in the presence of immunosuppressive factors enriched in solid tumors, suggesting their efficiency and safety for solid tumor immunotherapy [124**–**128].

In the case of triple-negative breast (TNBC) cancer, Liu et al. [129] showed that EGFR-CAR-NK cell(second-generation CAR) therapy may be a promising strategy for TNBC patients. Neither traditional cancer therapy nor conventional immunotherapy is effective for TNBC patients. EGFR-specific CAR-NK cells specifically trigger the lysis of TNBC cells in vitro. Or it can be used to treat TNBC patients showing enhanced expression of EGFR. Chen’s group [130] also demonstrated that EGFR-CAR NK-92 cells(second-generation CAR) increased cytolysis and IFNγ production in breast cancer cell lines MDA-MB-231, MDA-MB-468, and MCF-7 *(*in vitro*)*, and reduced tumor growth in tumor-bearing mice (in vivo). Hu [131] utilized a chimeric antigen receptor (CAR) approach, developed and tested tissue factor (TF)-CAR-NK(second-generation CAR), which co-expressed CD16, Fc receptor (FcγIII) mediated antibody-dependent cytotoxicity (ADCC). TF-CAR-NK cells can kill TNBC cells, and their efficacy can be enhanced with L-ICON ADCC in vitro. Furthermore, TF-CAR-NK cells were effective for the in vivo treatment of TNBC in both cell lines and patient tumor-derived xenograft mouse models. This study establishes evidence for the concept of targeting TF in CAR-NK immunotherapy as a novel target for effective treatment of TNBC.

### Gastric cancer (GC) and colon cancer (CRC)

Cao [132] engineered MSLN and CD19-targeted CAR NK-92 (MSLN and CD19-CAR NK) cells. The results showed that MSLN-CAR NK cells could specifically kill MSLN+ gastric cancer cells (N87, MKN-28 and AGS) in vitro, but had no effect on MSLN- cells (Huh-7). It was also observed that MSLN-CAR NK cells could effectively eliminate gastric cancer cells in subcutaneous and intraperitoneal tumor models. They also significantly prolong the survival of intraperitoneal tumor-bearing mice. More importantly, a strong antitumor effect and considerable NK cell infiltration were observed in MSLN-CAR-NK cell-treated patient-derived xenografts, which further demonstrated the therapeutic efficacy of MSLN-CAR-NK cell therapy in gastric cancer.

Xiao et al. [69] constructed NKG2D RNA CAR(second-generation CAR) by using the RNA electroporation method that provides transient expression of CAR, which significantly enhanced the cytolytic activity of NK cells against several solid tumor cell lines in vitro. Subsequently, three patients with metastatic colorectal cancer were treated with local infusion of NKG2D RNA-CAR NK cells. Reduced ascitic fluid production and a significant reduction in the number of tumor cells in ascitic fluid samples were observed in the first two patients receiving intraperitoneal infusion of low doses of CAR-NK cells. The third patient with liver tumor metastases received ultrasound-guided percutaneous injection followed by intraperitoneal infusion of CAR-NK cells. Rapid tumor regression in the liver region was observed by Doppler ultrasound imaging. Zhang et al. [133] constructed EpCAM-specific second-generation CAR and transduced them into NK-92 cells using lentiviral vectors. The resulting CAR-NK-92 cells could specifically recognize EpCAM-positive colorectal cancer cells, release cytokines (including IFN-γ, perforin, and granzyme B), and exhibit specific cytotoxicity in vitro. The anti-carcinoembryonic antigen (CEA)-CAR NK-92MI constructed by Shiozawa et al. [134] had significantly increased cytotoxicity against CEA-positive colon cancer cell lines.

### Liver cancer and pancreatic cancer

Liu [135] constructed a CAR structure targeting and recognizing c-MET antigen using lentivirus infection and demonstrated the specificity and effectiveness of c-MET targeting CAR-NK cell immunotherapy in the treatment of human liver cancer in vitro. Recently, Chen130 constructed GPC3-CAR-NK cells based on an affinity-enhanced antibody targeting GPC3 for the treatment of liver cancer patients. L3C7c-Fc was used to reverse the high level of soluble programmed death ligand 1 (sPD-L1) in HCC patients, enhancing the effect of this CAR-NK cell and providing experimental evidence for the development of subsequent liver cancer immunotherapy strategies.

For pancreatic ductal adenocarcinoma (PDAC), Lee et al. [136] found that folate receptor α (FRα) and death receptor 4 (DR4) were significantly overexpressed in PDAC cells. Co-expression of FRα and DR4/5 is associated with poorer clinical outcomes and is a potential target for biomolecular therapy. They reprogrammed allogeneic FRα CAR-NK cells to carry apoptosis-inducing ligands and redirected them to FRα and initiated DR45-mediated cancer-selective cell death in FRα and DR4/5-positive tumors. As a result, loading NK cells with redirected cytotoxic ligands led to significantly enhanced tumor-selective apoptosis. Da’s team [137] constructed anti-mesothelin (MSLN) CAR-NK-92 cells(second-generation CAR) through a transposon system, and verified that they showed more effective killing activity against pancreatic cancer cells. The anti-pancreatic cancer effect was further enhanced after co-culture with STING agonist cGAMP. Prostate stem cell antigen (PSCA)-CAR_s15 NK cells engineered by Teng et al. [138] exhibited significant tumor suppression.

### Renal cancer

At present, there are limited studies on the application of CAR-NK in urological tumors, and it remains a relatively underexplored field. Tumor necrosis factor-α-inducible protein 8-like 2 (TIPE2), encoded by TNFAIP8L2, is a newly discovered negative regulator of innate and acquired immunity and plays a key role in maintaining immune homeostasis. Studies have shown that the expression of TNFAIP8L2 is related to the poor prognosis of brain low-grade glioma (LGG), renal chromophobe cell carcinoma (chromophobe renal cell carcinoma) and renal clear cell carcinoma (Kidney renal clear cell carcinoma), and may become a CAR-NK Targets for the treatment of renal cancer [139]. Zhang [140] constructed the epidermal growth factor (EGFR-)-specific third-generation CAR through lentivirus. The specific killing ability of CAR-modified NK-92 cells (CAR-NK-92) on renal cell carcinoma (RCC) cell lines was confirmed in vitro. The synergistic effect of cabozantinib and EGFR-specific CAR-NK-92 cells was studied in vitro and in vivo, representing a breakthrough in CAR-NK therapy for kidney cancer.

## The clinical application potential of CAR-NK in solid tumors

The effectiveness of CAR-T in the treatment of hematological malignancies has been verified, but CAR-T cells will suffer from loss of target antigens, tumor heterogeneity, and immunosuppressive TME. Ineffective against solid tumors. NK cells also play an important role in the anti-tumor field, so the safer and more efficient CAR-NK have gradually attracted everyone’s attention and pursuit. NK cells modified by CAR structure can also theoretically efficiently recognize tumor cells and kill tumor cells by releasing killing mediators and inducing apoptosis of target cells. Compared with CAR-T in the treatment of solid tumors, first of all, CAR-NK is safer. Recently, CAR-NK immunotherapy has been confirmed in multiple clinical settings. Due to the limited lifetime of CAR-NK cells in circulation, the risk of side effects on normal tissues and graft-versus-host disease (GVHD) is relatively low [141]. Secondly, CAR-NK cells have dual anti-tumor activities. In addition to killing tumor target cells in the state of chimeric antigen receptors [142], CAR-NK cells themselves have anti-cancer effects and can be activated independently. NK cells without chimeric antigen receptors can kill cancer cells directly by releasing Perforin-1 and Granzyme or by death receptors, or by secreting cytokines and chemokines to activate immune cells such as T cells and B cells, and can also kill cancer cells by antibody dependent cell-mediated cytotoxicity (ADCC), which can better fight against tumor heterogeneity [143]. Thirdly, the low risk of rejection of NK cells allows CAR-NK cells to be generated from a variety of sources, including NK92 cell lines, peripheral blood mononuclear cells (PBMCs), cord blood (UCB), and induced pluripotent stem cells (iPSCs) [144]. Using advanced genetic engineering technology, NK cells can co-express other molecules with CARs, including cytokines, antibodies, proteases, etc., which can promote NK cell proliferation, transport and penetration into tumors. Fourthly, modification of NK cell signaling pathways (activation of NKG2D via DAP10 [145], knockdown of TIGIT downregulation of inhibitory NKG2A receptors [26]) can enhance NK cell cytotoxicity. Fifth, it can be modified against the suppressive TME: NK cells transduced with cord blood-derived TGF-β receptor II (DNRII) can resist the inhibitory effect of TGF-β in the TME, retaining its killing ability and Interferon-γ secretion [146]. Alternatively, the inhibitory PD1 signaling in the TME is converted to an activating one by chimeric co-stimulatory switching receptors (CCCRs) including the extracellular domain of PD1, the transmembrane and cytoplasmic domains of NKG2D, and the cytoplasmic domain 4-1BB of NKG2D signal, thereby reversing the immunosuppressive effect of PD1 [118]. Ng et al. [103]. enhanced the response of CAR-NK cells to tumor-secreted chemokines (IL-8 receptor CXCR1) by matching CAR-NK cells Inhibition of tumor migration and invasion.

The data in Table 1 is from the US Clinical Trials Registry (ClinicalTrials.gov). At present, there are 25 clinical trials of CAR-NK applied to solid tumors, one of which has published the results: NCT03415100 confirmed the strong lysis of NKG2D ligand-positive HCT116 human colorectal cancer cells by CAR-NK cells. Cell viability in the final product prior to infusion was greater than 90%. No serious adverse reactions (grade ≥ 3 adverse events) were found in any of the three patients. Only grade 1 cytokine release syndrome was observed, and no patient developed neurologic symptoms. The most common treatment-related adverse events reported included fever, fatigue, and anorexia. In order to truly apply CAR-NK therapy to clinical treatment in the future, we need to verify the basic experimental results of CAR-NK in different solid tumors introduced above in clinical trials, and make specific recommendations for different TME in the human body. Specific optimization, while also collecting side effects that occur during treatment. With the progress of these clinical trials, it is believed that more data will prove the clinical application potential of CAR-NK in solid tumors, and provide new treatment options for advanced complex solid tumors.

**Table. 1:** Summarized the current clinical trials using CAR-NK to treat solid tumors.

Row	Status	Gov Identifier	Study Title	Conditions	Interventions	Phase/*N*	Ages	Gender	Study Design	Cell Dosage	Outcome Measures	Locations
1	Recruiting	NCT05410717	Phase I/IIa Trial to Evaluate Safety and Preliminary Efficacy of CLDN6-CAR-NK in Patients With CLDN6-positive Advanced Solid Tumors	Stage IV Ovarian Cancer; Testis Cancer, Refractory; Endometrial Cancer Recurrent; CAR NK	Claudin6 targeting CAR-NK cells	Phase 1, Phase 2/*N* = 40	18 Years to 75 Years	All	Single Group Assignment/Open		Primary: Safety Secondary: ORR, DCR, DOR	The Second Affiliated Hospital of Guangzhou Medical University
2	Recruiting	NCT05213195	NKG2D CAR-NK Cell Therapy in Patients With Refractory Metastatic Colorectal Cancer	Refractory Metastatic Colorectal Cancer	NKG2D CAR-NK	Phase 1/*N* = 38	18 Years to 70 Years	All	Sequential Assignment/Open		Primary: Dose-Limiting Toxicity, Maximal Tolerable Dose Secondary: DOR, ORR, OS	The First Affiliated Hospital, Zhejiang University
3	Recruiting	NCT05528341	NKG2D-CAR-NK92 Cells Immunotherapy for Solid Tumors	Relapsed/Refractory Solid Tumor	NKG2D-CAR-NK92 cells	Phase 1/*N* = 20	18 Years to 75 Years	All	Single Group Assignment/Open	NKG2D-CAR-NK92 cells will be administered intravenously over 1 h. The starting dose of NKG2D-CAR-NK92 cells will be 0.5 × 10 ^ 6–2 × 10 ^ 6/kg, twice a week. The first evaluation of the efficacy after 3 weeks of treatment.	Primary: Safety, ORR Secondary: DCR, PFS, OS, Quality of Life Score	The first Affiliated Hospital of Xinxiang Medical University
4	Recruiting	NCT05776355	NKG2D CAR-NK & Ovarian Cancer	Ovarian Cancer	NKG2D CAR-NK	Not Applicable/*N* = 18	18 Years and older	Female	Single Group Assignment/Open		Primary: Dose-Limiting Toxicity, Maximal Tolerable Dose	Zhejiang Cancer Hospital
5	Recruiting	NCT05194709	Study of Anti-5T4 CAR-NK Cell Therapy in Advanced Solid Tumors	Advanced Solid Tumors	Anti-CAR-NK Cells	Early Phase 1/*N* = 40	18 Years to 80 Years	All	Single Group Assignment/Open	The administration of CAR-NK cell will be performed on day 1 and day 3 of each cycle (21 days). The first administration dose in the first cycle is 3.0 × 10 ^ 9 cells. If no adverse events were observed, the second administration dose in the first cycle would be 4.0 × 10 ^ 9 cells, and each administration dose in the second cycle and thereafter would be 4.0 × 10 ^ 9 cells.	Primary: safety and tolerability of anti-5T4 CAR-NK cells Secondary: ORR, PFS, OS, DCR, Cytokine release, Lymphocyte subtype	Wuxi People’s Hospital
6	Unknown	NCT03692637	Study of Anti-Mesothelin Car NK Cells in Epithelial Ovarian Cancer	Epithelial Ovarian Cancer	Anti-Mesothelin Car NK Cells	Early Phase 1/*N* = 30	18 Years to 70 Years	Female	Single Group Assignment/Open	Total dose of 0.5–3 million /kg cells will be administered at day 0	Primary: Occurrence of treatment related adverse events	No Contacts or Locations Provided
7	Recruiting	NCT05507593	Study of DLL3-CAR-NK Cells in the Treatment of Extensive Stage Small Cell Lung Cancer	SCLC, Extensive Stage	DLL3-CAR-NK cells	Phase 1/*N* = 18	18 Years to 75 Years	All	Sequential Assignment/Open	group A: 1.0 × 10 ^ 7, group B: 1.0 × 10 ^ 8, and group C: 1.0 × 10 ^ 9 DLL3-CAR-NK cells	Primary: DLT, MTD	Tianjin Medical University Cancer Institute and Hospital
8	Unknown	NCT03415100	Pilot Study of NKG2D-Ligand Targeted CAR-NK Cells in Patients With Metastatic Solid Tumours	Solid Tumour	Biological: CAR-NK cells targeting NKG2D ligands	Phase 1/*N* = 30	18 Years to 70 Years	All	Single Group Assignment/Open		Primary:Number of Adverse Events Secondary: Anti-tumour response due to CAR-NK cell infusions	Third Affiliated Hospital of Guangzhou Medical University
9	Unknown	NCT03940820	Clinical Research of ROBO1 Specific CAR-NK Cells on Patients With Solid Tumors	Solid Tumor	ROBO1 CAR-NK cells	Phase 1, Phase 2/*N* = 20	18 Years to 75 Years	All	Single Group Assignment/Open		Primary:Occurrence of treatment related adverse events as assessed by CTCAE v4.03	Radiation Therapy Department, Suzhou Cancer Center, Suzhou Hospital Affiliated to Nanjing Medical University
10	Recruiting	NCT04847466	Immunotherapy Combination: Irradiated PD-L1 CAR-NK Cells Plus Pembrolizumab Plus N-803 for Subjects With Recurrent/Metastatic Gastric or Head and Neck Cancer	Gastroesophageal Junction (GEJ) Cancers Advanced HNSCC	Drug: N-803 Drug: Pembrolizumab PD-L1 t-haNK	Phase 2/*N* = 55	18 Years and older	All	Single Group Assignment/Open	PD-L1 CAR NK cells (2 × 109) will be administered by IV infusion over approximately 30 min every week. After the week 6 treatment, these cells will be given every 2 weeks.	Primary: CR + PR Secondary:PFS, DOR, safety and tolerability	National Institutes of Health Clinical Center Bethesda, Maryland, United States
11	Not yet recruiting NEW	NCT05845502	Single-arm, Open-label Clinical Study of SZ003 in the Treatment of Advanced Hepatocellular Carcinoma	Advanced Hepatocellular Carcinoma	SZ003 CAR-NK	Not Applicable/*N* = 12	18 Years to 80 Years	All	Single Group Assignment/Open	SZ003 CAR-NK In the escalation study, the minimum initial dose was 5.0 × 10 ^ 6 cells, then increased to 1.0 × 10 ^ 8, 2.0 × 10 ^ 8 and 5.0 × 10 ^ 8 cells. The infusion is given every 2 weeks.	Primary: Number of Adverse Events, ORR, OS	Shantou University Medical College; Guangdong ProCapZoom Biosciences Co., Ltd.
12	Unknown	NCT03941457	Clinical Research of ROBO1 Specific BiCAR-NK Cells on Patients With Pancreatic Cancer	Pancreatic Cancer	BiCAR-NK cells (ROBO1 CAR-NK cells)	Phase 1, Phase 2/*N* = 9	18 Years to 75 Years	All	Single Group Assignment/Open		Primary: Occurrence of treatment related adverse events as assessed by CTCAE v4.03	Department of Radiology, Shanghai Ruijin Hospital
13	Unknown	NCT03931720	Clinical Research of ROBO1 Specific BiCAR-NK/T Cells on Patients With Malignant Tumor	Malignant Tumor	BiCAR-NK/T cells (ROBO1 CAR-NK/T cells)	Phase 1, Phase 2/*N* = 20	18 Years to 75 Years	All	Single Group Assignment/Open		Primary: Occurrence of treatment related adverse events as assessed by CTCAE v4.04	Department of Oncology, Suzhou Kowloon Hospital, Shanghai Jiaotong University School of Medicine
14	Not yet recruiting	NCT05686720	Single-arm, Open-label Clinical Study of SZ011 in the Treatment of Advanced Triple Negative Breast Cancer	Advanced Triple Negative Breast Cancer	SZ011 CAR-NK	Early Phase 1/*N* = 12	18 Years to 80 Years	Female	Single Group Assignment/Open	the minimum initial dose was 5.0 × 10 ^ 6 cells, then increased to 5.0 × 10 ^ 7 and 2.0 × 10 ^ 8 cells. The infusion is given every 2 weeks.	Primary: Number of Adverse Events, ORR Secondary: PFS, DOR, DCR,OS	First Affiliated Hospital of Shantou University Medical College Guangdong ProCapZoom Biosciences Co., Ltd.
15	Recruiting	NCT03692663	Study of Anti-PSMA CAR NK Cell (TABP EIC) in Metastatic Castration-Resistant Prostate Cancer	Metastatic Castration-resistant Prostate Cancer	TABP EIC; Cyclophosphamide2023-6-5; fludarabine	Early Phase 1/*N* = 9	18 Years and older	All	Single Group Assignment/Open	A single dose of 0.5, 10, and 30 million TABP EIC will be iv administered at D0, D7, and D14.	Primary: Occurrence of treatment related adverse events as assessed by CTCAE v5.0 Secnodary:he pharmacokinetic analysis of TABP EIC, The proportion of patients with a decrease in PSA levels from baseline, PFS,Time to clinical progression	Tianjin pepole’s hosptial
16	Not yet recruiting	NCT05856643	Single-arm, Open-label Clinical Study of SZ011 in the Treatment of Ovarian Epithelial Carcinoma	Ovarian Epithelial Carcinoma	Drug: SZ011 CAR-NK	Early Phase 1/*N* = 12	18 Years to 80 Years	Female	Single Group Assignment/Open	In the escalation study, the minimum initial dose was 5.0 × 10 ^ 6 cells, then increased to 1.0 × 10 ^ 8, 2.0 × 10 ^ 8 and 5.0 × 10 ^ 8 cells. The infusion is given every 2 weeks.	Primary: Number of Adverse Events, ORR, OS, PFS	Shantou University Medical College
17	Recruiting	NCT05248048	NKG2D CAR-T Cells to Treat Patients With Previously Treated Liver Metastatic Colorectal Cancer	Refractory Metastatic Colorectal Cancer	Biological: CAR-T infusion	Early Phase 1/*N* = 9	18 Years to 75 Years	All	Single Group Assignment/Open		Primary: DLT, MTD Secondary: ORR, OS	The Third Affiliated Hospital of Guangzhou Medical University
18	Recruiting	NCT03383978	Intracranial Injection of NK-92/5.28.z Cells in Combination With Intravenous Ezabenlimab in Patients With Recurrent HER2-positive Glioblastoma	Glioblastoma	NK-92/5.28.z; Ezabenlimab	Phase 1, *N* = 42	18 Years and older	All	Single Group Assignment/Open	Intracranial application of NK-92/5.28.z, 1x10E7-1x10E8 intravenous infusion of Ezabenlimab 240 mg q 3 weeks	Primary: Number of participants with treatment-related adverse events, MTD or MFD for NK-92/5.28.z, Period of detectability of NK-92/5.28.z cells in blood and cerebrospinal fluid (CSF) during the first 24 weeks after NK-92/5.28.z application with qPCR, qPCR detection of NK-92/5.28.z in blood or CSF. Cytokine profile in the blood and the cerebrospinal fluid Secondary: immune response., ORR, PFS, OS	Neurochirurgische Klinik, Universitätsmedizin Mannheim; Neurochirurgische Klinik, Universitätsmedizin Mainz; Neurochirurgische Klinik, Universitätsmedizin Mainz; Johann W. Goethe University Hospital, Senckenberg Institute of Neurooncology
19	Recruiting	NCT05703854	Study of CAR.70-engineered IL15-transduced Cord Blood-derived NK Cells in Conjunction With Lymphodepleting Chemotherapy for the Management of Advanced Renal Cell Carcinoma, Mesothelioma and Osteosarcoma	Advanced Renal Cell Carcinoma;Advanced Mesothelioma; Advanced Osteosarcoma	CAR.70/IL15-transduced CB-derived NK cells; Fludarabine phosphate	Phase 1, Phase 2/*N* = 50	18 Years to 80 Years	All	Single Group Assignment/Open		Incidence of Adverse Events	M D Anderson Cancer Center
20	Unknown	NCT02839954	CAR-pNK Cell Immunotherapy in MUC1 Positive Relapsed or Refractory Solid Tumor	Hepatocellular Carcinoma; Non-small Cell; Lung Cancer; Pancreatic Carcinoma; Triple-Negative Invasive Breast Carcinoma; Malignant Glioma of Brain; Colorectal Carcinoma; Gastric Carcinoma	Anti-MUC1 CAR-pNK cells	Phase 1, Phase 2/*N* = 10	18 Years and older	All	Single Group Assignment/Open		Primary: Adverse events attributed to the administration of the anti-MUC1 CAR-pNK cells, Determine the toxicity profile of the MUC1 targeted CAR-pNK cells with Common Toxicity Criteria for Adverse Effects Secondary: ORR	PersonGen BioTherapeutics (Suzhou) Co., Ltd.
21	Recruiting	NCT05137275	Study of Anti-5T4 CAR-raNK Cell Therapy in Locally Advanced or Metastatic Solid Tumors	Locally Advanced or Metastatic Solid Tumors	Anti-5T4 CAR-raNK Cells	Early Phase 1/*N* = 56	18 Years to 80 Years	All	Single Group Assignment/Open	In the 3 + 3 dose escalation study, the minimum initial dose is 3.0 × 10 ^ 9 cells and then escalate to 6.0 × 10 ^ 9 and 9.0 × 10 ^ 9 cells. Every 21 days is one cycle, and intravenous infusion is performed on day 1 and day 3\8 of each cycle. In dose extension study, the initial dose will be determined by RP2D determined by the results of dose escalation study, and the other intervention methods are consistent.	Primary: DLTs, AEs, ORR, DCR, DOR, PFS, OS Secondary: The number of CAR-raNK cells, Cytokine release, Lymphocyte subtype, Anti-CAR antibodies	Shanghai East Hospital
22	Recruiting	NCT05143151	CD276-targeted Chimeric Antigen Receptor T Cells in Treatment With Advanced Pancreatic Cancer	Advanced Pancreatic Carcinoma	CD276 CAR-T cells		18 Years to 75 Years	All	Single Group Assignment/Open		Primary: ORR Secondary: OS	
23	Recruiting	NCT03882840	Induced-T Cell Like NK Cellular Immunotherapy for Cancer Lack of MHC-I	Anti-cancer Cell Immunotherapy T Cell and NK Cell	NK cell therapy	Phase 1, Phase 2/*N* = 10	18 Years and older	All	Single Group Assignment/Open		Primary: The safety and tolerance of the ITNK cell immunotherapy Secondary: Percent of Patients with best response as either complete remission or partial remission.	The Second Affiliated Hospital of Guangzhou Medical University
24	Enrolling by invitation	NCT03656705	CCCR-NK92 Cells Immunotherapy for Non-small Cell Lung Carcinoma	Non-small Cell Lung Cancer	CCCR-NK92 cells	Phase 1, *N* = 12	18 Years to 75 Years	All	Single Group Assignment/Open	CCCR-NK92 cells will be administered intravenously over 1 h. The starting dose of CCCR-NK92 cells will be 1 × 10e7-1 × 10e8, twice a week. The first evaluation of the efficacy after 3 weeks of treatment.	Primary: Number of participants with adverse events, ORR. Secondary: DCR, PFS, OS	The first Affiliated Hospital of Xinxiang Medical University
25	Ongoing	ChiCTR2100048100	Safety Study of autologous MESO CAR NK cells in the treatment of refractory epithelial ovarian carcinoma	Epithelial ovarian carcinoma	MESO CAR NK	Phase I	18 Years to 70 Years	Female	Single arm/Open		Primary: safety	Zhuhai People’s Hospital

Including the status, conditions, interferences, phase, age/gender, locations, and other information of the experiment.

## Conclusion

The clinical application of CAR-NK against solid tumors is largely influenced by TME. Hypoxia, acidic environment, high adenosine environment, NK cell surface receptors, TGF-β, IL, and exosomes all affect the anti-tumor activity of CAR-NK. It can inhibit tumor cell immune escape by modifying the intrinsic pathway, combating tumor heterogeneity, antagonizing inhibitory TME, and improving the ability of CAR-NK cells to infiltrate tumor tissue. Despite ongoing clinical trials, there remains a limited understanding of the mechanisms underlying CAR-NK efficacy in solid tumors, particularly in liver cancer, cholangiocarcinoma, and urological tumors. While some trials are underway, many are in the preliminary stage with small sample sizes, and limitations are relatively large. The true clinical application of CAR-NK therapy requires in-depth research and experimentation.

In the 21st century, the incidence of tumors has been steadily increasing, yet our understanding of them remains limited. Effective treatments for advanced solid tumors are lacking, leading to a low survival rate for patients. Immunotherapy, particularly CAR-NK therapy, holds promise for patients with advanced malignant solid tumors. Further exploration is needed to enhance the efficacy of CAR-NK therapy, ultimately maximizing the survival rate and improving the quality of life for these patients.
